# Structural Brain Abnormalities, Diagnostic Approaches, and Treatment Strategies in Vertigo: A Case-Control Study

**DOI:** 10.3390/neurolint17090146

**Published:** 2025-09-10

**Authors:** Klaudia Széphelyi, Szilvia Kóra, Gergely Orsi, József Tollár

**Affiliations:** 1 Doctoral School of Health Sciences, Faculty of Health Sciences, University of Pécs, 7622 Pécs, Hungary; silviasabokora@gmail.com (S.K.); tollarjozsef86@gmail.com (J.T.); 2 Faculty of Health Sciences and Social Studies, Department of Theoretical Health Sciences and Health Management, University of Szeged, 6726 Szeged, Hungary; 3 Department of Neurology, Medical School, University of Pécs, 7622 Pécs, Hungary; gergo.orsi@gmail.com; 4 HUN-REN-PTE Clinical Neuroscience MR Research Group, Hungarian Research Network, 7622 Pécs, Hungary; 5 Somogy County Móricz Kaposi Teaching Hospital, 7400 Kaposvár, Hungary; 6 Digital Development Center, Széchenyi István University, 9026 Győr, Hungary; 7 Department of Otorhinolaryngology-Head and Neck Surgery, University of Pécs Medical School, 7622 Pécs, Hungary; 8 János Bolyai Research Scholarship of the Hungarian Academy of Science, 1051 Budapest, Hungary; 9 Faculty of Health Sciences, University of Pécs, 7622 Pécs, Hungary

**Keywords:** vertigo, MRI, white matter lesions, circle of Willis, sinusitis, BPPV, lifestyle modifications, betahistine

## Abstract

**Background/Objectives:** Dizziness is a frequent medical complaint with neurological, otolaryngological, and psychological origins. Imaging studies such as CT (Computer Tomography), cervical X-rays, and ultrasound aid diagnosis, while MRI (Magnetic Resonance Imaging) is crucial for detecting brain abnormalities. Our purpose is to identify structural brain changes associated with vertigo, assess pre-MRI diagnostic approaches, and evaluate treatment strategies. **Methods:** A case-control study of 232 vertigo patients and 232 controls analyzed MRI findings, pre-MRI examinations, symptoms, and treatments. Statistical comparisons were performed using chi-square and *t*-tests (*p* < 0.05). **Results**: White matter lesions, lacunar infarcts, Circle of Willis variations, and sinusitis were significantly more frequent in vertigo patients (*p* < 0.05). Pre-MRI diagnostics frequently identified atherosclerosis (ultrasound) and spondylosis (X-ray). Common symptoms included headache, imbalance, and visual disturbances. The most frequent post-MRI diagnosis was Benign Paroxysmal Positional Vertigo (BPPV). Treatments included lifestyle modifications, physical therapy (e.g., Epley maneuver), and pharmacological therapies such as betahistine. **Conclusions**: MRI revealed structural brain changes linked to vertigo. Pre-MRI assessments are essential for ruling out vascular and musculoskeletal causes. A multidisciplinary treatment approach is recommended. **Trial Registration:** This study was registered in ClinicalTrials.gov with the trial registration number NCT06848712 on 22 February 2025.

## 1. Introduction

Vertigo is one of the most common complaints in medical practice. It is associated with various underlying conditions, including neurological, otolaryngological, and psychological disorders. The prevalence of dizziness in the adult population is in the range of 20–30%, and it is particularly common among the elderly, significantly impacting their quality of life and daily functioning. Among those over 65 years of age, the prevalence of dizziness reaches 30%, while in individuals over 75, it increases to 50%, highlighting the public health significance of this issue in aging populations [1,2].

The etiology of dizziness is often multifactorial, involving degenerative changes in the vestibular system and neurological disorders, as well as circulatory and musculoskeletal conditions. The most frequently diagnosed causes include benign paroxysmal positional vertigo (BPPV), Ménière’s disease, vestibular neuritis, and central nervous system vascular diseases [3,4].

Dizziness is often accompanied by other symptoms, such as headache, imbalance, psychological issues, and nausea/vomiting, which are also critical for establishing an accurate diagnosis. Detailed examination of these symptoms, as well as preliminary imaging studies such as CT (Computer Tomography), cervical spine X-rays, and carotid/vertebral artery ultrasound, aids in precise diagnosis and targeted therapeutic decisions. However, it is important to recognize that routine MRI (Magnetic Resonance Imaging) often fails to identify specific causes of dizziness, as structural abnormalities—such as white matter lesions—are common in both symptomatic and asymptomatic populations [5,6].

MRI is currently one of the most effective imaging modalities for examining the brain. MRI enables the assessment of soft tissues, including clear visualization of gray and white matter, ventricles, blood vessels, and nerves (such as the VIII. cranial nerve—the vestibulocochlear nerve, damage of which can result in dizziness). The standard protocol includes the following sequences: sagittal T1-weighted, coronal FLAIR (Fluid-Attenuated Inversion Recovery), axial T2-weighted, axial DWI (Diffusion Weighted Imaging), and axial SWI (Susceptibility Weighted Imaging). When necessary, post-contrast 3D sagittal T1-weighted sequences are performed. For dizziness, a 3D axial balanced steady-state gradient echo sequence (FIESTA/TrueFISP/balanced-FFE on GE, Siemens, and Philips, respectively) is always acquired to examine the inner ear, while for headaches, TOF (Time-of-Flight) sequences are added to the protocol [7,8,9].

This study aims to identify the brain structural abnormalities in vertigo that can be diagnosed through brain MRI. Additionally, it provides a detailed overview of diagnostic procedures related to dizziness, with a particular focus on pre-MRI investigations, and analyzes the most common symptoms and therapeutic approaches. Through this analysis, the study seeks to advance the understanding and management of dizziness in clinical practice.

## 2. Materials and Methods

### 2.1. Participants and Design

Patients were enrolled from the outpatient clinic of Szent Margit Hospital between September 2020 and December 2022.

In the control group, vertigo was not reported as a symptom. Exclusion criteria included the presence of any neurological disease or complaint in the patient’s medical history. A total of 232 participants were enrolled. The mean age of the participants was 56.77 ± 17.14 years (age range 18–91 years). There were no clinical evaluations in the control group, only the MRI diagnosis was examined.

In the vertigo group, the inclusion criteria were that the patient reported dizziness as a symptom and that the attending physician had requested a brain MRI examination. Patients were not examined in the emergency setting for acute vertigo; instead, they suffered from longer-standing or recurrent dizziness. Exclusion criteria included the presence of any neurological disease or complaint in the patient’s medical history (other than vertigo or dizziness). A total of 232 participants were enrolled. The mean age of the participants was 59.41 ± 17.43 years (age range 19–93 years). We examined the medical history, including symptoms other than dizziness and the examinations conducted prior to the MRI. The most common abnormalities observed in the MRI results were categorized, including cortical atrophy, white matter lesions, vascular lesions, lacunar lesions, vascular encephalopathy, Circle of Willis variation, and sinusitis. Additionally, we collected the final diagnoses and treatments administered following the MRI.

### 2.2. MRI Protocol

The MRI parameters are summarized in Table 1. The brain MRI protocol consisted of the following measurements:

- sagittal T1-weighted
- coronal FLAIR
- axial T2-weighted
- axial DWI
- axial SWI (Susceptibility Weighted Imaging)
- axial TOF
- axial 3D FIESTA (this is a supplementary measurement for the inner ear, which is always included in the protocol in cases of vertigo)
- optional: contrast enhanced 3D sagittal T1-weighted.

### 2.3. Outcomes

**Figure 1:**
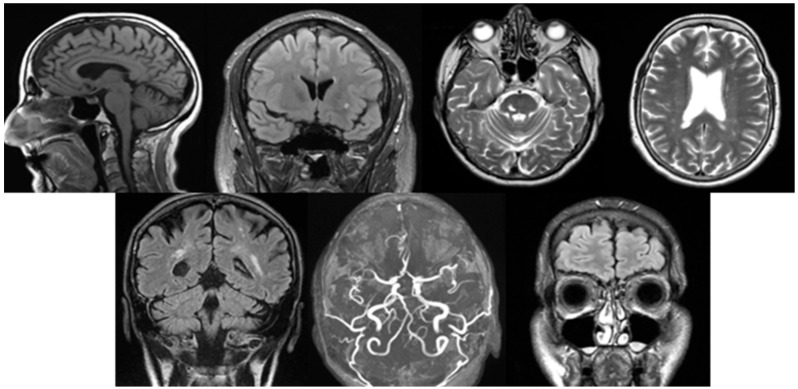
From left to right in the first row are cortical atrophy (sagittal T1-weighted, cortical thickness and widening of the sulci), white matter lesion (coronal FLAIR, hyperintense periventricular changes), vascular lesions (axial T2-weighted, focal hyperintensity), and lacunar lesion (axial T2-weighted, hyperintense deep white matter changes), and in the second row are vascular encephalopathy (coronal FLAIR, diffuse white matter hyperintensities), circle of Willis variation (axial TOF-MRA, anatomical variation in the arterial configuration), sinusitis (coronal FLAIR, mucosal thickening and sinus opacification).

The primary outcomes are the structural changes described during MRI. Throughout the study, all findings were systematically evaluated by a neuroradiologist. To diagnose various pathologies, neurologists in Hungary apply local professional guidelines and the recommendations of the National Health Professional College. The most common pathologies were diagnosed based on the following criteria (Figure 1).

- Cortical atrophy: The Scheltens scale aids in the standardized grading of atrophy severity. It is characterized by a reduction in cortical thickness, widening of the sulci, and dilation of the ventricular system, particularly the lateral ventricles. These changes are typically observed on T1-weighted and FLAIR images, where thinning of the cortical structures is evident [10].
- White matter lesions: The Fazekas scale and the STRIVE (Standards for Reporting Vascular Changes on Neuroimaging) criteria are the most commonly used standards. White matter lesions are identified on FLAIR and T2-weighted images as bright (hyperintense) areas located in the periventricular or deep white matter. These may appear as discrete spots or confluent regions, typically indicating small vessel disease [11].
- Vascular lesions: In Hungary, the European Stroke Organization Guidelines serve as the primary reference. MR angiography is routinely used to assist in the diagnosis of vascular abnormalities. On DWI, acute ischemic lesions appear as hyperintense signals. T1/T2-weighted and MRA (Magnetic Resonance Aniography) images can identify stenosis, occlusions, or vascular wall abnormalities. In cases of haemorrhage, hemosiderin deposits can be detected using SWI techniques [12].
- Lacunar lesions: The STRIVE criteria assist in identifying these lesions. They are characterized by small (<15 mm) round or oval hyperintense areas on T2-weighted/FLAIR images. Common locations include the thalamus, basal ganglia, and internal capsule. On T1-weighted images, they appear as hypointense signals [11].
- Vascular encephalopathy: The Hungarian Stroke Guidelines and the recommendations of the MSKT (Hungarian Stroke Consensus Council), along with the application of the Fazekas scale, are essential for evaluation. Diffuse white matter hyperintensities are observed on FLAIR images. Subcortical infarcts, dilated perivascular spaces, or signs of microvascular disease may also be present. Cortical and subcortical atrophy is frequently associated with ventricular enlargement [13].
- Circle of Willis variation: The evaluation of the Circle of Willis focuses on assessing its completeness, anatomical variations, and flow characteristics. TOF-MRA is commonly used for visualization, allowing the detection of stenosis, occlusion, aneurysms, and collateral flow patterns. Variations or abnormalities are identified based on flow signal uniformity and vessel connections [14].
- Sinusitis: The ACR (American College of Radiology) Appropriateness Criteria and the Lund-Mackay scoring system are utilized. Sinus opacification, mucosal thickening, or fluid levels are identified on T2-weighted images. In acute inflammation, high-intensity signals and fluid levels are observed on T2. In chronic sinusitis, findings include thickened bony walls, fibrosis, or the presence of mucoceles [15].

Secondary outcomes are other symptoms appearing in addition to vertigo, the examinations preceding the MRI, diagnosis after MRI, and treatments applied after the MRI examination.

### 2.4. Statistical Analyses

Demographic characteristics of the groups were compared to prove that age and gender distribution were similar. Age was analyzed using an independent samples *t*-test, while gender distribution was assessed with a chi-square test. Normality was assessed using the Shapiro–Wilk test, and the data followed a normal distribution.

Descriptive statistics were used to summarize the baseline characteristics. Data are reported as mean ± SD (Standard Deviation).

To evaluate differences in MRI findings between the control and vertigo groups, chi-square tests were conducted for cortical atrophy, white matter lesions, vascular lesions, lacunar infarcts, vascular encephalopathy, Circle of Willis variation, and sinusitis/sinus abnormalities.

Since the examinations preceding the MRI, MRI-related diagnoses, and therapies were not assessed in the control group, we focused on analyzing the frequencies of secondary findings statistically to provide insights into their distribution and potential relevance.

The significance level was set at *p* < 0.05 for all analyses.

Statistical calculations were conducted using IBM SPSS Statistics for Windows, Version 26.0., IBM Corp., Armonk, NY, USA.

### 2.5. Ethical Considerations

This study was registered at ClinicalTrials.gov under the identifier NCT06848712 on 22 February 2025. The study was approved by the Regional Ethical Committee of Szent Margit Hospital (IG/296-1/2025/2) on 4 February 2025. The study was conducted according to the World Medical Association Declaration of Helsinki. As it was a retrospective study, informed consent was waived in accordance with institutional guidelines. Data collection complied with GDPR (General Data Protection Regulation) and patient confidentiality regulations.

## 3. Results

### 3.1. Baseline Characteristics

Table 2 shows the descriptive data in the vertigo group. The study involved 68 male and 164 female participants, with an average age of 59.41 (±17.43) years. The participants’ ages ranged from 19 to 93 years. Examinations preceding the MRI, other symptoms, and MRI results are discussed in detail in the outcome results.

Table 3 shows the descriptive data in the control group. The study involved 76 male and 156 female participants, with an average age of 56.77 (±17.43) years. The participants’ ages ranged from 18 to 91 years. MRI results are discussed in detail in the outcome results.

No significant differences were found between the control and vertigo groups for age (t(462) = −1.646, *p* = 0.100) or gender distribution (χ^2^(1, *n*= 464) = 0.644, *p* = 0.422).

### 3.2. Primary Outcome

Of the enrolled subjects, 75% had positive MRI findings (structural changes). The MRI results are summarized in Table 4.

In the vertigo group, white matter lesions (39.2%) were described the most frequently as a result of brain MRI, followed by cortical atrophy (25.9%) and vascular encephalopathy (31.5%).

**Figure 2:**
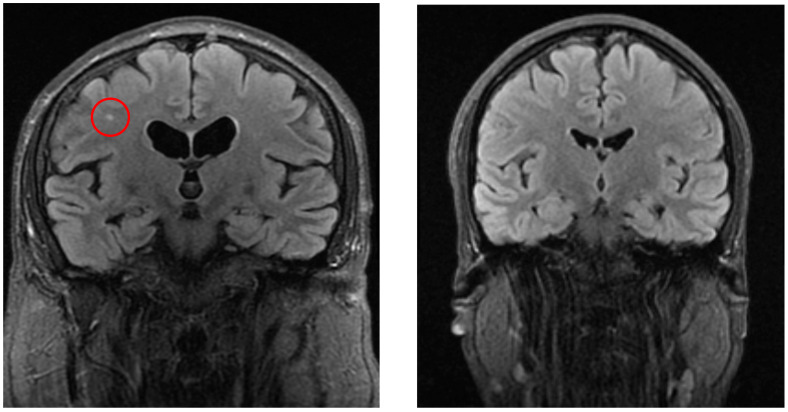
The left image (coronal FLAIR) shows a white matter lesion (red circle) in a patient from the vertigo group, whereas the right image (coronal FLAIR) demonstrates a control patient without such lesions.

Significant differences were detected in white matter lesions (*p* = 0.001, see Figure 2), lacunar lesions (*p* = 0.047), Circle of Willis variation (*p* = 0.03), and sinusitis/sinus abnormalities (*p* = 0.005). Other MRI findings, including cortical atrophy, vascular lesion, and vascular encephalopathy, did not show statistically significant differences (*p* > 0.05).

**Figure 3:**
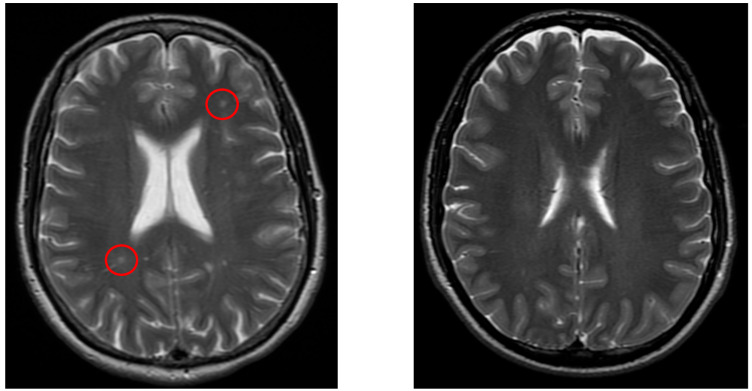
The left image (axial T2-weighted) shows lacunar lesions (red circle) in a patient from the vertigo group, whereas the right image (axial T2-weighted) demonstrates a control patient without such lesions.

Figure 3 demonstrates a typical lacunar infarct detected in the vertigo group, in contrast with controls.

**Figure 4:**
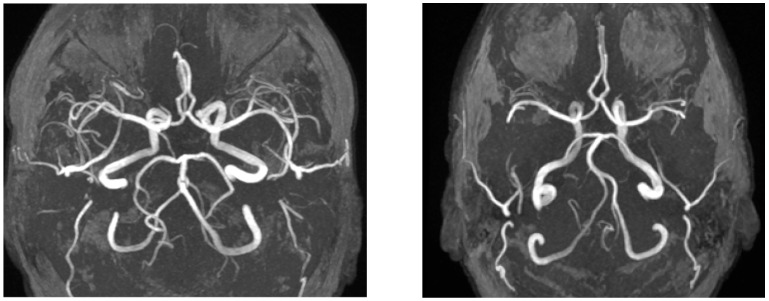
The left image (axial TOF-MRA) shows a variation of the basilar artery in a patient from the vertigo group, whereas the right image (axial TOF-MRA) demonstrates a control patient with a normal Circle of Willis.

Representative images of Circle of Willis variations are shown in Figure 4.

**Figure 5:**
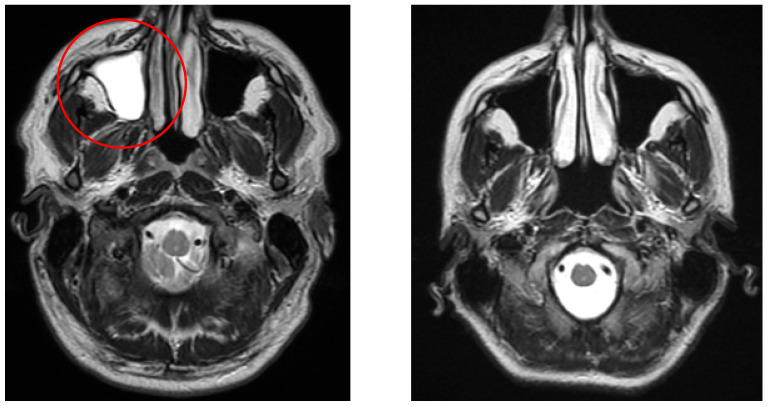
The left image (axial T2-weighted) shows an extensive inflammation in the right maxillary sinus (red circle) in a patient from the vertigo group, whereas the right image (axial T2-weighted) demonstrates a control patient with a clear sinus.

As illustrated in Figure 5, patients from the vertigo group often showed sinus abnormalities, whereas no such abnormalities were observed in the control group.

### 3.3. Secondary Outcome

Table 5 presents the types of examinations performed prior to MRI, the number of cases for each examination type, and the number of positive findings. Among the 31 CT scans, 18 showed positive results. For carotis/vertebralis ultrasound, 75 cases were conducted, with 34 yielding positive findings. The most common finding in carotis/vertebralis ultrasound examinations was evidence of atherosclerosis (*n* = 26). Cervical spine X-rays were performed in 23 cases, with 21 positive results. The most common finding in cervical spine X-rays was spondylosis (*n* = 13). Additionally, 36 cases involved other types of examinations, of which 29 were positive. Other imaging examinations included for example cervical spine MRI, paranasal sinus X-rays, and EEG (Electroencephalogram).

Table 6 summarizes the symptoms experienced by patients, highlighting that headache was the most common symptom (24.14%), followed by imbalance (15.5%). Vomiting and nausea were reported in 11.2%, tinnitus in 8.6%, and psychological symptoms in 5.2%. Among the other symptoms, the most common ones were vision-related issues (*n* = 15) and numbness (*n* = 11).

The diagnoses after MRI are summarized in Table 7. In 44 patients, no final diagnosis was provided. Among the remaining 188 cases, the most frequently diagnosed conditions were BPPV (34.5%) and Vertebrobasilar Syndrome (VBS, 11.6%).

The applied treatments are summarized in Table 8. The most common interventions included lifestyle advice (21.1%) and physical training (15.01%). Physical training includes interventions such as the Epley maneuver and spinal exercises. Pharmacological treatments were less frequent, with cognitive enhancers (10.3%), anxiolytics, hypnotics, and antidepressants (6.5%), and anticoagulants (7.3%) being the most common. Other treatments, such as lipid-lowering medications, herbal medicines, and betahistin, were used in 2–6% of cases. Rare interventions included antimigraine medications (1.7%) and steroids (0.4%).

## 4. Discussion

This study investigates the underlying structural brain changes associated with vertigo.

Our primary outcome revealed that white matter lesions, lacunar lesions, variations of the Circle of Willis, and sinusitis were significantly more prevalent in patients presenting with vertigo compared to the control group. Midbrain white matter lesions have been observed more frequently in patients with dizziness. In our study, we did not examine the precise localization of white matter lesions. In another study, similar to ours, white matter lesions were also found to be significantly more frequent in the dizziness group. This research also highlighted that non-specific white matter lesions were more common [16].

Brain MRI examinations were frequently preceded by carotid/vertebral artery ultrasound, where the findings suggested that atherosclerosis could be a contributing factor to dizziness. Among cervical spine X-rays, the majority showed abnormal findings, with spondylosis being the most common diagnosis [17].

In addition to dizziness, the most frequently reported symptoms were headache, imbalance, visual disturbances, and numbness. In some publications, tinnitus and hearing loss are mentioned as symptoms accompanying dizziness, in contrast to the symptoms observed in our study [18].

Following the MRI results, the treating physicians most frequently diagnosed patients with BPPV. Other publications have also found BPPV to be one of the most common peripheral vestibular disorders. Similar to our results, in Whitman’s article, the most common causes of dizziness included BPPV, vestibular migraine, anxiety disorders, orthostatic hypotension, and medication side effects [19]. Although not observed in our cohort, it is important to mention vestibular schwannoma, which can also present with vertigo. Vestibular schwannoma is a benign tumor arising from Schwann cells of the vestibular nerve, most commonly from its inferior division [20]. For example, in the case report by Joon Yong Park and Chang-Hee Kim, a vestibular schwannoma caused vertigo to a 57-year-old patient [21].

In the treatment of dizziness, lifestyle modifications and various physical exercises were the most commonly recommended interventions, including techniques such as the Epley maneuver and spinal exercises. Among pharmacological therapies, the use of betahistine and anxiolytics, hypnotics, and antidepressants were the most widespread, aiming to address both the vestibular symptoms and the psychological burden associated with chronic vertigo. Other studies have also identified physical exercises targeting BPPV, such as repositioning maneuvers, and the use of betahistine as the most common and effective therapeutic approaches [22].

The nature of dizziness was not recorded by referring physicians in 155 cases, preventing us from examining this aspect in detail. However, other studies have identified significant associations between dizziness and the structural characteristics of specific brain regions [23].

## 5. Limitation

A limitation of our study is that we did not examine the precise localization of structural brain abnormalities. Additionally, since we only collected MRI diagnoses for the control group, we could only report frequencies for other parameters we analyzed (such as prior examinations and treatments). Another limitation is that we did not conduct a detailed examination of the variations in the Circle of Willis.

## 6. Conclusions

In addition to dizziness, the most commonly reported symptoms are headache, imbalance, and visual disturbances. Prior to MRI, vertebral/carotid artery ultrasound is often indicated to rule out atherosclerosis, while cervical spine X-rays are used to exclude spondylosis. Based on MRI findings, in addition to various white matter lesions, malformations of the Circle of Willis and inflammations of the paranasal sinuses may also contribute to dizziness symptoms. In terms of treatment, alongside symptomatic therapies (e.g., BPPV exercises, cervical spine exercises, Epley maneuver, and betahistine), addressing lifestyle-related triggers (e.g., using antidepressants or providing lifestyle counseling) is also essential.

## Figures and Tables

**Table 1:** MRI pulse sequences parameters.

	Sequence Type	TR ^1^	TE ^2^	TI ^3^	FOV ^4^	Matrix ^5^	FS ^6^	Scan Time ^7^	NEX ^8^	b-Value ^9^	Bandwith ^10^
** Sagittal T1 **	FSE ^11^	577	12,34	-	240	512 × 512	N	3,5	31.25	-	1
** Axial T2 **	FSE	7580	86.5	-	260	512 × 512	N	3.5	62.5	-	1.5
** Coronal T2 **	FLAIR	9000	120	2580	240	256 × 256	N	3.5	70	-	1
** Axial DWI **	SE EPI ^12^	7551	83.1	-	260	256 × 256	N	2.5	250	20/1000	1
** Axial SWI **	GRE ^13^	73.7	47.07	-	260	512 × 512	N	3	41.67	-	1
** Axial TOF **	SPGR ^14^	26	6.8	-	220	512 × 512	Y	3.5	250	-	1
** +c Sagittal 3D T1 **	FSPGR ^15^	6.884	2.06	-	240	512 × 512	N	3.5	83.33	-	1
** Axial 3D FIESTA **	FIESTA GRE ^16^	6.90	2.54	-	270	512 × 512	N	3.5	125	-	1

^1^ TR—Repetition Time (ms); ^2^ TE—Echo Time (ms); ^3^ TI—Inversion Time (ms); ^4^ FOV—Field of View (mm); ^5^ Matrix (pixel); ^6^ FS—Fat Saturation (N-no, Y-yes); ^7^ Scan Time (min); ^8^ NEX—Number of Excitations; ^9^ b-value (s/mm^2^); ^10^ Bandwidth (kHz); ^11^ FSE—Fast Spin Echo; ^12^ SE EPI—Spin Echo Echo Planar Imaging; ^13^ GRE—Gradient Echo; ^14^ SPGR—Spoiled Gradient Echo; ^15^ FSPGR—Fast Spoiled Gradient Echo; ^16^ FIESTA GRE—Fast Imaging Employing Steady-State Acquisition Gradient Echo.

**Table 2:** Descriptive characteristics of the patients with vertigo.

Variable	Count or Mean	±SD
Number of subjects (males)	232 (68)	
Age	59.41 years	17.43 years
Examinations preceding the MRI, *n*		
Carotis/vertebralis US (Ultrasound)	75 (32%)	
CT	31 (13%)	
Cervical spine X-ray	23 (9%)	
Other	36 (15%)	
Other symptoms, *n*		
Headache	56 (24%)	
Imbalance	36 (15%)	
Vomiting, nausea	26 (11%)	
Tinnitus	20 (9%)	
Psychological	12 (5%)	
Other	71 (31%)	
MRI results, *n*		
White matte lesion	91 (39%)	
Vascular encephalopathy	73 (31%)	
Cortical atrophy	60 (26%)	
Circle of Willis variation	51 (22%)	
Sinusitis	39 (17%)	
Lacunar lesion	31 (13%)	
Vascular lesion	13 (6%)	

**Table 3:** Descriptive characteristics of the control subjects.

Variable	Count or Mean	±SD
Number of subjects (males)	232 (76)	
Age	56.77 years	17.43 years
MRI results, *n*		
Vascular encephalopathy	62 (27%)	
White matte lesion	58 (25%)	
Cortical atrophy	52 (22%)	
Lacunar lesion	47 (20%)	
Circle of Willis variation	33 (14%)	
Sinusitis	19 (8%)	
Vascular lesion	8 (3, 4%)	

**Table 4:** Incidence of observed structural changes.

Results	Vertigo Group (%)	Control Group (%)
White matter lesion	39.2	25
Vascular encephalopathy	31.5	26.7
Cortical atrophy	25.9	22.4
Circle of Willis variation	22	14.2
Sinusitis	16.8	8.2
Lacunar lesion	13.4	20.3
Vascular lesion	5.6	3.4

**Table 5:** Examinations preceding the MRI.

Examinations	Number of Cases	Positive Cases
Carotis/vertebralis US	75	34 (45%)
CT	31	18 (58%)
Cervical spine X-ray	23	21 (91%)
Other	36	29 (81%)

**Table 6:** Other symptoms.

Symptoms	%
Headache	24.14
Imbalance	15.5
Vomiting, nausea	11.2
Tinnitus	8.6
Psychological	5.2
Other	30.6

**Table 7:** Final diagnosis after MRI.

Final Diagnosis	%
BPPV	34.5
VBS	11.6
Psychological	5.6
Encephalopathy	4.7
Sclerosis	2.6
Brain infarction	4.3
Headache	4.3
Sinusitis	2.2
Central Balance Syndrome	2.2
Not specified	19
Other	9.1

**Table 8:** Treatments.

Treatments	%	Treatments	%
Lifestyle advance	21.1	Anti-inflammatory	2.2
Physical training	15.01	Lipid-lowering medications	2.2
Cognitive enhancers	10.3	Antimigraine medications	1.7
Anticoagulant	7.3	Antiepileptics	1.3
Anxiolytics, hypnotics, antidepressants	6.5	Antiparkinson medications	0.9
Betahistin	6	Steroid	0,4
Herbal medicine	3	Other drugs	6
Vitamins and minerals	3	Other non—pharmacological	5.17

## Data Availability

Authors are committed to making the data available upon request.

## Abbreviations

The following abbreviations are used in this manuscript:

BPPV	Benign Paroxysmal Positional Vertigo
CT	Computed Tomography
MRI	Magnetic Resonance Imaging
FLAIR	Fluid-Attenuated Inversion Recovery
DWI	Diffusion Weighted Imaging
SWI	Susceptibility Weighted Imaging
TOF	Time-of-Flight
TR	Repetition Time
ms	milliseconds
TE	Echo Time
TI	Inversion Time
FOV	Field of View
mm	millimeters
FS	Fat Saturation
min	minutes
NEX	Number of Excitations
s/mm^2^	seconds per square millimeter
kHz	kilohertz
FSE	Fast Spin Echo
SE EPI	Spin Echo Echo Planar Imaging
GRE	Gradient Echo
SPGR	Spoiled Gradient Echo
FSPGR	Fast Spoiled Gradient Echo
FIESTA GRE	Fast Imaging Employing Steady-State Acquisition Gradient Echo
STRIVE	Standards for Reporting Vascular Changes on Neuroimaging
MRA	Magnetic Resonance Angiography
MSKT	Magyar Stroke Konszenzus Tanács (Hungarian Stroke Consensus Panel)
ACR	American College of Radiology
GDPR	General Data Protection Regulation
US	Ultrasound
EEG	Electroencephalogram
VBS	Vertebrobasilar Syndrome
